# Decreased CX3CL1 Levels in the Cerebrospinal Fluid of Patients With Alzheimer’s Disease

**DOI:** 10.3389/fnins.2018.00609

**Published:** 2018-09-07

**Authors:** Juan R. Perea, Alberto Lleó, Daniel Alcolea, Juan Fortea, Jesús Ávila, Marta Bolós

**Affiliations:** ^1^Department of Molecular Neuropathology, Centro de Biología Molecular Severo Ochoa, Consejo Superior de Investigaciones Científicas, Madrid, Spain; ^2^Network Center for Biomedical Research on Neurodegenerative Diseases, Madrid, Spain; ^3^Memory Unit, Department of Neurology, Hospital de la Santa Creu i Sant Pau, Biomedical Research Institute Sant Pau, Universitat Autònoma de Barcelona, Barcelona, Spain

**Keywords:** CX3CL1, Alzheimer’s disease, tau, microglia, human CSF, inflammation

## Abstract

Alzheimer’s disease (AD) is a neurodegenerative disease characterized by the presence of neurofibrillary tangles, constituted by tau protein, and plaques formed by amyloid-beta protein. The disease courses with high neural damage, which leads to memory loss and death. Here we analyzed the presence of CX3CL1, a chemokine expressed by neurons, in cerebrospinal fluid (CSF) samples from control subjects and patients with mild cognitive impairment and AD dementia. CX3CL1 was decreased in the CSF of AD dementia patients compared to control subjects. However, there was not difference in plasma samples from the same subjects.

## Introduction

Alzheimer’s disease (AD) is the most common form of dementia (Alzheimer’s Association, 2016). It is characterized by the accumulation of extracellular plaques of amyloid-beta (Aβ) protein (Selkoe, 1997) and intracellular neurofibrillary tangles formed by hyperphosphorylated and/or aggregated tau protein (Sjogren et al., 2001). In addition, AD is accompanied by prominent neuronal and synaptic loss (Bondareff et al., 1987). Together, these hallmarks lead to the characteristic neurodegeneration observed in this disease. Assays for the detection of proteins related to the main pathological characteristics of the disease, such as neurofibrillary tangles, amyloid plaques, and neuronal death have been developed. In this regard, the prevalence of the biomarkers identified was then tested in cerebrospinal fluid (CSF) samples from AD patients and controls (Blennow and Zetterberg, 2018). CSF of the former showed a lower amount of Aβ42 and a higher level of tau (Motter et al., 1995; Galasko et al., 1998; Andreasen et al., 2003).

Inflammation is associated with AD (Bolos et al., 2017b). However, whether inflammation is a cause or a consequence of the development or progression of the disease is unknown. Inflammation in the central nervous system (CNS) is regulated mainly by microglia (Thomas, 1992). To maintain brain homeostasis, these cells, which are in charge of CNS surveillance, establish continuous communication with other types of brain cells, such as neurons and astrocytes, through the expression, interaction and secretion of chemokines, among other molecules (Mennicken et al., 1999). Chemokines have several functions in the CNS, including the mediation of cell to cell communication, during development and in adulthood (Martin and Delarasse, 2018). CX3 chemokine ligand 1 (CX3CL1), also named fractalkine, is expressed by neurons as membrane-bound and soluble forms (Bazan et al., 1997). This chemokine interacts with the CX3 chemokine receptor 1 (CX3CR1) present in microglia (Imai et al., 1997; Maciejewski-Lenoir et al., 1999). Therefore, there is a direct communication axis between neurons and microglia performed by the CX3CL1/CX3CR1 tandem (Harrison et al., 1998; Sheridan and Murphy, 2013). Furthermore, neurons modulate microglial function through the CX3CL1/CX3CR1 axis (Mecca et al., 2018). It has been suggested that this axis is impaired with aging and in neurological diseases such as AD (Sheridan and Murphy, 2013; Bolos et al., 2017a).

Given the presence of inflammation in AD, we studied whether CX3CL1 is altered with the progression of the disease. To this end, we used CSF and plasma samples from control subjects, patients with mild cognitive impairment (MCI), and patients with AD dementia.

## Material and Methods

### Study Participants

We analyzed 42 CSF derived from control, MCI and AD subjects and 28 plasma samples derived from the same control and AD donors. Participants were recruited in the Hospital de la Santa Creu i Sant Pau, Barcelona, as part of the cohort of the Sant Pau Initiative on Neurodegeneration (SPIN) (Alcolea D, submitted). Ethic committee number 16/2013.

Participants had a diagnosis of MCI due to AD, AD dementia with evidence of the pathophysiological process (Frisoni et al., 2011; McKhann et al., 2011), or were cognitively normal controls. All participants had CSF and blood samples available, which were obtained at the same time.

All AD patients had abnormal values of Aβ_42_, total tau (tau) and phospho-tau (ptau) and all controls had normal values (**Table 1**). All participants underwent a complete neuropsychological evaluation using a previously published protocol (Dubois et al., 2007). Furthermore, they all gave written informed consent to participate in the study. The local ethics committee at Sant Pau Hospital approved the study. Participants were selected from a group of donors that were free of inflammatory disorders and had not drug therapy that confounding effect on inflammation. The absence of inflammation was confirmed by blood analysis from the blood extracted from donors.

**Table 1:** Demographics and CSF biomarker levels of the participant.

Group	Cases (*N*)	Age (Mean years ± SD)	Gender (female/male)	MMSE ± SD	CSF ABETA42 (pg/ml) ± SD	CSF P-TAU (pg/ml) ± SD	CSF TAU (pg/ml) ± SD	CX3CL1 (ng/ml) ± SD
Control	14	64 ± 2.92	7/8	29 ± 0.88	802 ± 218	59 ± 42	244 ± 46	0.31 ± 0.05
MCI	14	70 ± 3.52^∗∗∗^	9/5	26 ± 2.95^∗∗^	438 ± 58^∗∗∗^	112 ± 51^∗^	761 ± 374 n.s	0.28 ± 0.07
AD	14	68 ± 4.18^∗^	9/5	21.5 ± 3.5	453 ± 101^∗∗∗^	109 ± 46^∗^	762 ± 377 n.s	0.166 ± 0.05^∗∗∗^

*Three groups of samples: C, control; MCI, mild cognitive impairment; AD, Alzheimer’s disease, with 14 cases (N) in each group, were analyzed. The average age, gender, memory test (MMSE), amount of CSF Aβ42, CSF ptau, CSF tau and CSF CX3CL1 are detailed in the table. Results are expressed as average concentration ± standard deviation (SD). Statistics of the measures are related to control: ^∗^p ≤ 0.05, ^∗∗^p ≤ 0.01, and ^∗∗∗^p ≤ 0.001.*

### CSF Analysis

Core AD biomarkers [Aβ_42_, total tau (tau) and phospho-tau (ptau)] were analyzed at the Hospital Sant Pau using commercially available ELISA kits (Innotest^TM^ β-Amyloid_1-42_, Innotest^TM^hTAU Ag, and Innotest^TM^ Phospho-tau_181P_; Fujirebio-Europe, Ghent, Belgium), following the manufacturer’s instructions. The cut-off values for normal core AD biomarkers were Aβ_42_ > 550 pg/ml, tau < 350 pg/ml, and ptau < 61 pg/ml (Alcolea et al., 2015).

### Human CX3CL1 ELISA

The total amount of CX3CL1 present in CSF derived from control, MCI and AD dementia samples or from plasma derived from control and AD dementia samples was analyzed by ELISA (R&D Systems, United States, catalog number DCX310), following the manufacturer’s instructions. Each sample was tested in duplicate. The results were expressed as concentration of CX3CL1 (ng/ml). The mean of the coefficient of variation (CV) of sample duplicates is 6.19% and the CV between-plates is 9.69%. The mean of the limit of detection (LOD) of this ELISA is 0.018 ng/ml (range: 0.006–0.072 ng/ml), as indicated by the manufacturer’s instructions as “mean of minimum detectable dose of human fractalkine.” Samples analyzed were over the LOD. In addition, the lower limit of quantification (LLOQ) was calculated as blank + 9 SD of the blank, obtained a LLOQ = 0.063 and CV = 18.24%. Only samples which values were > LLOQ were analyzed.

### Statistical Analysis

The Kolmogorov–Smirnov test was used to test the normality of the sample distribution. Data were analyzed by a Student’s *t*-test, because the distribution was normal, or by one-way analysis of variance. *Post hoc* comparisons were analyzed using Tukey’s multiple comparison test. Differences were considered statistically significant when the probability, *p*, of the null hypothesis was ≤ 0.05. Data are presented as the means ± SE.

## Results

Forty-two CSF samples were analyzed in this study (**Table 1**). Demographic and CSF markers are detailed in **Table 1**.

### CX3CL1 in CSF Decreases in AD

**FIGURE 1:**
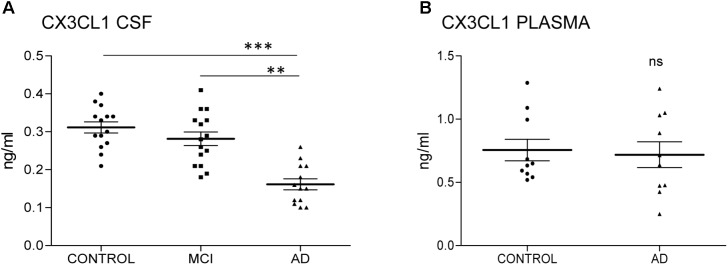
CX3CL1 decreases in the CSF of AD patients. *N* = 14 samples per group: control (C), mild cognitive impairment (MCI), and Alzheimer’s disease (AD) were analyzed by ELISA of CX3CL1. Samples were run in duplicate. The concentration of CX3CL1 was analyzed in CSF **(A)** and plasma samples **(B)**. CX3CL1 decreased in the CSF of AD compared to control and MCI **(A)**. However, no difference was observed in the concentration of CX3CL1 in plasma samples **(B)**. Bars show means ± SE. ^∗∗^*p* ≤ 0.01, ^∗∗∗^*p* ≤ 0.001.

We have previously shown that the amount of total CX3CL1 increases in AD brains with the progression of the disease (Bolos et al., 2017a). To analyze whether this difference in CSF, we measured the amount of CX3CL1 by ELISA. Three groups of CSF samples (**Table 1**): control, MCI and AD dementia were tested. One sample from the AD group was eliminated for statistical proposes because was lower than the LLOQ. There was no difference between controls and MCI (C = 0.31 ± 0.05 ng/ml vs. MCI = 0.28 ± 0.07 ng/ml; *p* = 0.198) (**Figure 1A** and **Table 1**). However, a marked decrease in the concentration of CX3CL1 was observed between MCI and AD dementia (MCI = 0.28 ± 0.07 ng/ml vs. AD = 0.166 ± 0.05 ng/ml; ^∗∗^*p* < 0.01). Furthermore, a clear decrease in CX3CL1 concentration was also found when control and AD dementia samples were compared (C = 0.31 ± 0.05 ng/ml vs. AD = 0.166 ± 0.05 ng/ml; ^∗∗∗^*p* < 0.0001). Therefore, a reduction of CX3CL1 was observed in AD dementia.

Next, the amount of CX3CL1 was measured in plasma. Because the amount of CX3CL1 in CSF didn’t change in MCI compared to control subjects, we focus the measure on samples from control subjects and AD dementia patients. No differences between groups were detected (C = 0.75 ± 0.027 ng/ml vs. AD = 0.72 ± 0.032 ng/ml; *p* = 0.782) (**Figure 1B**). These results confirmed that the variation of CX3CL1derives from changes within the CNS and not in the periphery.

## Discussion

Here, we show for the first time that neuronal CX3CL1 is decreased in the CSF samples from AD dementia patients compared to those from control subjects. Furthermore, the concentration of CX3CL1 is reduced in AD dementia compared to MCI samples but not in MCI compared to C samples. Therefore, decreased levels of CX3CL1 are more evident in advanced stages (dementia).

CX3CL1 is a chemokine expressed mainly in neurons in the CNS (Mennicken et al., 1999). In these cells, it is present as a membrane-bound protein and, after its excision by alpha-secretases such as ADAM10 (Hundhausen et al., 2003) and ADAM17 (Garton et al., 2001), among others, it is secreted as a soluble form. This effect is also observed for the amyloid precursor protein (APP), which is cleaved by ADAM10 within the Aβ domain, thus preventing Aβ generation (Kuhn et al., 2010). Soluble CX3CL1 has a chemoattractive effect for monocytes, natural killer cells, and lymphocyte cells (Bazan et al., 1997; Chapman et al., 2000). However, the exact role of each isoform remains unknown. Both isoforms of the chemokine mediate their effects by interaction with the CX3CR1 receptor, which is expressed exclusively by microglia (Harrison et al., 1998). The interaction between CX3CL1 and CX3CR1 has both beneficial and detrimental consequences throughout the activation of various pathways within microglia (Imai et al., 1997; Re and Przedborski, 2006). It has been proposed that the binding of CX3CL1 to CX3CR1 maintains microglia in a resting state, thereby decreasing the production of pro-inflammatory cytokines and preventing the activation response by these cells (Biber et al., 2007). Therefore, correct functionality of the CX3CL1/CX3CR1 axis is crucial for the maintenance of brain homeostasis, and especially for dealing with microglia-mediated inflammation in the CNS (Chen et al., 2016). In this regard, we have recently detected an imbalance in the CX3CL1/CX3CR1 axis at later stages of AD dementia (Bolos et al., 2017a). Furthermore, this imbalance has been reported in other diseases (Sheridan and Murphy, 2013). In addition, both CX3CL1 and CX3CR1 increase in later stages of AD dementia (Bolos et al., 2017a). Here, we report a decrease in CX3CL1 in the CSF of AD dementia patients compared to that of control subjects. This decreased CX3CL1 corresponds to the soluble form of the protein (∼70 kDa in western blot, data not shown), which is the form that is secreted. In this regard, ADAM10 activity decreases in AD dementia (Kim et al., 2009; Mukda et al., 2016; Manzine et al., 2018), thereby allowing the cleavage of APP by beta and gamma-secretases, thus leading to Aβ peptide, which is the toxic protein accumulated in AD dementia brains (Endres and Deller, 2017). Therefore, we hypothesize that, during the progression of AD dementia, the expression of a membrane-bound and less soluble form of CX3CL1 in the brain increases as a result of reduced ADAM10 activity. This increase is reflected by a decrease in the secretion of soluble CX3CL1 into the CSF of AD dementia patients compared to controls and MCI subjects.

Changes in CX3CL1 concentration have been found in other diseases that course with inflammation. For example, soluble CX3CL1 levels are increased in plasma and CSF of patients with lupus erythematosus (Guo et al., 2017). In addition, plasma CX3CL1 levels are augmented in individuals with type-2 diabetes (Sindhu et al., 2017). In this regard, we did not find any difference in this parameter in the plasma samples from AD dementia patients compared to those from control subjects. Recently, other proteins related to microglia and astrocytes, such as TREM2 (Suarez-Calvet et al., 2016) and YKL-40, have been put forward as potential biomarkers for AD (Alcolea et al., 2014, 2015, 2017). Furthermore, and in the same context as our work, a recent study has described the clinical use of inflammatory CSF protein markers in AD dementia (Brosseron et al., 2018). In that study, the authors analyzed several cytokines and other factors related to inflammation in the CSF of AD dementia patients. They found changes when compared with control samples. Although we did not find any difference in the levels of CX3CL1 CSF between MCI and C samples, a marked difference was detected at the advanced stage of dementia.

Decreased levels of Aβ42 and increased amounts of tau and ptau in the CSF of AD dementia patients are considered biomarkers for the disease (Jack et al., 2018). In addition, changes in CX3CL1 were detected in AD compared to MCI CSF samples and not in control compared to MCI. Overall, our results show that changes in the concentration of CX3CL1 could be a novel target to be explored in the context of inflammation and AD.

## Author Contributions

MB and JÁ conceived and designed the study. AL, DA, and JF provided the samples. MB and JP collected the data. MB, JÁ, and AL analyzed the data. MB, JP, AL, JF, and JÁ drafted the manuscript. All authors read and approved the final manuscript.

## Conflict of Interest Statement

The authors declare that the research was conducted in the absence of any commercial or financial relationships that could be construed as a potential conflict of interest.
